# Intrinsic coating morphology modulates acute drug transfer in drug-coated balloon therapy

**DOI:** 10.1038/s41598-019-43095-9

**Published:** 2019-05-02

**Authors:** Gary H. Chang, Dara A. Azar, Chimera Lyle, Vipul C. Chitalia, Tarek Shazly, Vijaya B. Kolachalama

**Affiliations:** 1 Section of Computational Biomedicine, Department of Medicine, Boston University School of Medicine, Boston, MA 02118 USA; 2 Biomedical Engineering Program, College of Engineering and Computing, University of South Carolina, Columbia, SC 29208 USA; 3 Renal Section, Department of Medicine, Boston University School of Medicine, Boston, MA 02118 USA; 4 Whitaker Cardiovascular Institute, Boston University School of Medicine, Boston, MA 02118 USA; 5 Hariri Institute for Computing and Computational Science & Engineering, Boston University, Boston, MA 02215 USA; 6 Department of Medicine, Veterans Affairs Boston Healthcare System, Boston, MA USA

**Keywords:** Interventional cardiology, Translational research

## Abstract

The hallmark of drug-coated balloon (DCB) therapy for the treatment of peripheral vascular disease is that it allows for reopening of the narrowed lumen and local drug delivery without the need for a permanent indwelling metal implant such as a stent. Current DCB designs rely on transferring drugs such as paclitaxel to the arterial vessel using a variety of biocompatible excipients coated on the balloons. Inherent procedural challenges, along with limited understanding of the interactions between the coating and the artery, interactions between the coating and the balloon as well as site-specific differences, have led to DCB designs with poor drug delivery efficiency. Our study is focused on two clinically significant DCB excipients, urea and shellac, and uses uniaxial mechanical testing, scanning electron microscopy (SEM), and biophysical modeling based on classic Hertz theory to elucidate how coating microstructure governs the transmission of forces at the coating-artery interface. SEM revealed shellac-based coatings to contain spherical-shaped microstructural elements whereas urea-based coatings contained conical-shaped microstructural elements. Our model based on Hertz theory showed that the interactions between these intrinsic coating elements with the arterial wall were fundamentally different, even when the same external force was applied by the balloon on the arterial wall. Using two orthogonal cell-based assays, our study also found differential viability when endothelial cells were exposed to titrated concentrations of urea and shellac, further highlighting the need to maximize coating transfer efficiency in the context of DCB therapies. Our results underscore the significance of the excipient in DCB design and suggest that coating microstructure modulates acute drug transfer during device deployment.

## Introduction

Peripheral arterial disease (PAD) was estimated to affect over 200 million people around the world in the preceding decade and this number is increasing continuously^1,2^. Endovascular interventions such as percutaneous transluminal angioplasty (PTA) with or without stenting are among the preferred choices for the treatment of PAD. Recent randomized controlled clinical trials have demonstrated the superiority of drug-coated balloon (DCB) therapy when compared to PTA alone, at least in terms of improved patency and reduced target lesion revascularization^3–7^. In all these studies, paclitaxel (PTX) was the drug of choice but each device differed in terms of the drug dosing, the selection of the excipient and the overall coating formulation. For example, two DCBs that have recently obtained regulatory approval within the United States have 3.5 µg/mm^2^ of PTX with urea as the excipient (IN.PACT Admiral, Medtronic, Santa Rosa, CA, USA), and 2 µg/mm^2^ of PTX with a mixture of polysorbate and sorbitol as the excipient (Lutonix DCB, C. R. Bard, New Hope, MN, USA), respectively. Additionally, few DCBs that are undergoing clinical trials as well as the ones in development also use PTX as the drug, (e.g. Stellarex DCB, Spectranetics, Colorado Springs, CO, USA containing 2 µg/mm^2^ of PTX with polyethylene glycol as the excipient). During the endovascular procedure, the belief is that an optimal coating enables efficient transfer of drug from the balloon to the artery, with concomitant adherence to the arterial wall. The adhered coating is then expected to serve as a sustained drug source, leading to a second phase of drug delivery to the arterial wall. Studies have shown that only a small portion (<8%) of the coating gets transferred during balloon inflation^8^, and almost 90% of the delivered drug is lost within about 24–48 hours^9^. These numbers point to the poor efficiency of drug delivery from balloon catheters. Hence, there is a need to better understand the factors influencing delivery efficiency and what causes these persistent limitations^10,11^.

We sought to examine the interactions between the balloon coating and the arterial wall on multiple scales to better understand device-dependent arterial pharmacokinetics. We selected PTX as the drug along with urea and shellac as the excipients for the balloon coating in our experimental study. While a balloon catheter using urea as an excipient is already in clinical use within the United States (IN.PACT Admiral, Medtronic) for the treatment of PAD^6,7^, a DCB device with shellac as the excipient (DIOR II, Eurocor) is approved in the European Union for coronary applications^12^. First, we performed a series of bench-top experiments to estimate coating-specific mechanical behavior that allowed us to characterize the contact between the balloon coating and the arterial wall. Using these parameters, we developed a biophysical model to quantify the interfacial mechanics as a function of the intrinsic shape of the coating microstructure and the force applied by the balloon on the arterial wall. Developed metrics of interfacial mechanics were then associated with experimental measurements of arterial drug transfer. To further quantify excipient-based differences among potential DCBs, we examined cultured human endothelial cell viability when treated with increasing concentrations of urea and shellac. While the mechanical tests and modeling relate to essential device function at the time of DCB deployment, the latter studies are relevant to the notion of increasing coating adherence/retention to serve as a sustained drug source. Taken together, obtained results underscore the importance of coating composition as a determinant factor of DCB efficiency and suggest that coating microstructure modulates the acute drug delivery from these devices.

## Materials and Methods

### Preparation of PTX-excipient coated and control films

**Figure 1:**
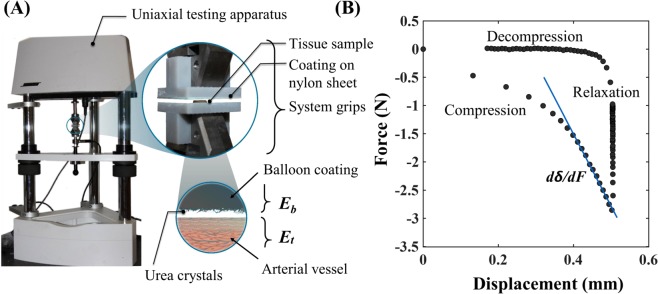
(**A**) Mechanical testing setup customized for uniaxial compression testing. Schematic of the 2-element test system containing the arterial vessel and the drug-coated balloon is also shown. (**B**) A typical force-displacement (FD) curve that results from a mechanical test. Data resulting from the FD curve was used to estimate compliance values of the test construct.

We developed balloon coatings with PTX and urea (1:1), as well as with PTX and shellac (1:1), using a micropipetting-coating method. Briefly, urea and shellac, 5–20% w/v, were dissolved in ethanol-200 at ~200 rpm for 4–6 hours. PTX solution was prepared in ethanol-200 at 10 mg/mL and added dropwise to the excipient solutions to generate several (5–20%) w/v mixtures. Each solution was then micropipetted on a sheet of balloon material (Nylon-12), and was kept at room temperature for 6 hours, which resulted in a coated surface with 3 µg/mm^2^ PTX with either excipient. The dried films were cut in 25 × 25 mm squares and glued to a rigid 3D-printed testing block, creating flat test elements for uniaxial mechanical testing (Fig. 1A).

### Mechanical characterization of coated balloon in isolation

The compressive mechanical response of coated balloons was measured using a mechanical testing system (Bose ElectroForce 5270) configured for uniaxial testing. The created test elements were placed between two flat plates, and subjected to a compressive displacement (0.005 mm/sec) until the force response was 1, 2 or 3N. Once the target force was reached, the compression plates were held at the same position for a dwell time of 60 s, following by unloading of the sample at the same displacement rate. Sample force and displacement data (Fig. 1B), were continuously recorded at an acquisition rate of 20 s^−1^ using a system-integrated software (Wintest).

**Figure 2:**
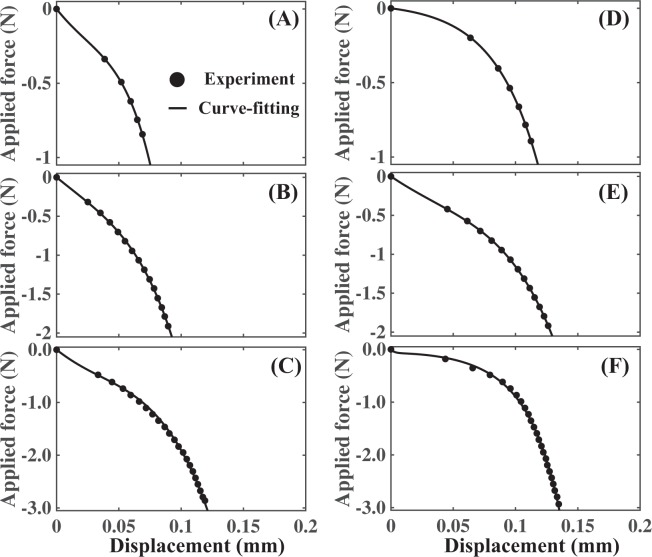
Force-displacement curves. (**A**–**C**) Average force-displacement curves generated from the compression test. Curve-fitting results for the shellac coated balloon with application force of 1N (**A**), 2N (**B**) and 3N (**C**) are shown. (**D**–**F**) Average force-displacement curves for the compression test, followed by curve-fitting results for the urea coated balloon with application force of 1N (**D**), 2N (**E**) and 3N (**F**) are shown.

To facilitate calculation of the reduced modulus of the coated balloon, the resultant force-displacement data were fit to a two-term exponential model, defined as:

1 $$F=\sum _{i=1}^{2}\,\exp ({\alpha }_{i}{\delta }_{b}),$$

where *F* is the recorded compressive force, *δ*_*b*_ is the displacement, and *α*_*i*_ are the parameters for the exponential model (Fig. 2). The reduced elastic modulus of the coated balloon then becomes:

2 $$\frac{1}{{E}_{b}^{\ast }}=\sqrt{A}\frac{d{\delta }_{b}}{dF},$$

where

3 $$\frac{d{\delta }_{b}}{dF}={[\sum _{i=1}^{2}{\alpha }_{i}\exp ({\alpha }_{i}{\delta }_{b})]}^{-1}$$

### Mechanical characterization of coated balloon in contact with arterial vessel

During balloon inflation, the coating contacts and directly interacts with the arterial wall. To facilitate procedural simulation with our coated balloon test elements, we created analogous flat arterial tissue elements from porcine femoral arteries obtained from a local abattoir immediately following animal sacrifice (8–12-month-old, 75–125 lbs, male American Yorkshire pigs). We configured these two elements – the coated balloon and arterial tissue sample – in series to enable uniaxial compression testing (Fig. 1A). The same loading-dwell-unloading protocol as described above was applied to this 2-element system. Obtained data allowed relation of the overall compliance of this 2-element system (*dδ*_0_/*dF*) to ${E}_{b}^{\ast }$ (obtained above; Fig. 1B), and the reduced modulus of the arterial wall (${E}_{t}^{\ast }$), as:

4 $$\frac{1}{{E}_{t}^{\ast }}=\sqrt{A}\frac{d{\delta }_{0}}{dF}-\frac{1}{\,{E}_{b}^{\ast }},$$

where *δ*_0_ is the total displacement of the 2-element system, *F* is the recorded compressive force, *A* is the overall contact area between the balloon coating and the arterial wall, ${E}_{b}^{\ast }$ and ${E}_{t}^{\ast }$ are the reduced moduli of the balloon and arterial wall, respectively^13,14^.

### Scanning electron microscopy

**Figure 3:**
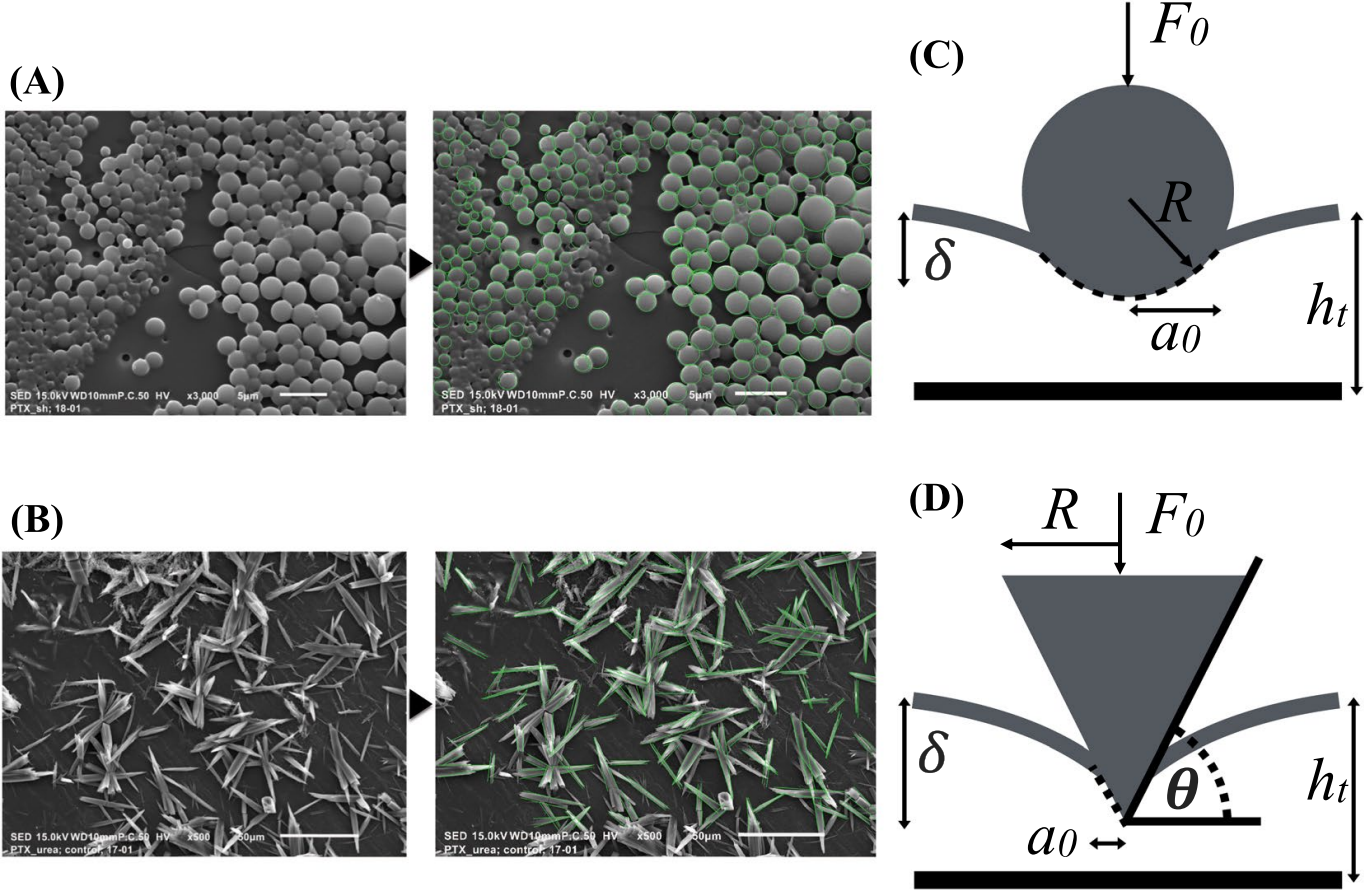
Intrinsic shape of the balloon coatings. SEM imaging revealed a spherical structure for shellac (**A**), and conical structure for urea (**B**). Contact mechanics models were developed based on Hertz theory by considering a spherical element for shellac (**C**) and a conical element for urea (**D**).

Variable pressure scanning electron microscopy (SEM) (Tescan Vega-3 SBU) was used to assess the surface microstructure of the coated balloons. Obtained images were processed to measure the average intrinsic shape of the coatings (Matlab, MathWorks, Natick, MA). Each SEM image was first converted to grayscale and then binarized. The ‘regionprops’ feature was used to read all the objects within the binarized image and several properties including mean perimeter and mean contact angle were estimated that defined the intrinsic shape of shellac and urea microstructures, respectively (Fig. 3A,B). For shellac, we observed a spherical microstructure, whereas for urea, we noticed a micro needle-like structure with a polygonal cross-section along the length of the needle, thus representing a conical contact with the arterial surface. Note that the conical contact assumption was made for cases when the micro needle was lying flat on the arterial surface or when the tip of the micro needle was in contact with the arterial surface.

### Contact mechanics model

We modeled the interactions between the balloon coating and the artery during balloon angioplasty as an elastic contact problem using the classic Hertz theory. The surface of the balloon coating has a unique microstructure characterized by its intrinsic shape and asperity. For PTX-shellac, we assumed that when the spherical microstructures come in contact with the arterial vessel, they result in an elastic deformation within the region of the point of contact. This contact region is smaller than the surface area of the spherical element and is defined using (Fig. 3C):

5 $${a}_{0}=\sqrt{\delta R},$$

where *a*_0_ is the contact radius, *δ* is the indentation depth, and *R* is the radius of the intrinsic spherical element^15,16^. The resulting force applied by the spherical element on the arterial wall then becomes:

6 $${F}_{0}=\frac{4}{3}{E}_{t}^{\ast }{R}^{1/2}{\delta }^{3/2},$$

where ${E}_{t}^{\ast }$ is the reduced elastic modulus of the arterial wall. While the force applied results in the elastic interaction between the spherical coating element and the arterial wall, only the contact region experiences this force. Thus, the distribution of this force over a smaller region is quantified by the mean contact pressure $\bar{P}={F}_{0}/(\pi {a}_{0}^{2})$. For PTX-shellac, this becomes:

7 $$\bar{P}=\frac{4}{3\pi }{E}_{t}^{\ast }\sqrt{\frac{\delta }{R}}.$$

For the case of PTX-urea, the observed needle-like structures were considered as conical structures with sharp facet angles (Fig. 3B), where the contact radius is defined as (Fig. 3D):

8 $${a}_{0}=\frac{2\delta }{\pi \,\tan \,\theta },$$

where *θ* is the angle (assumed as 30°) between the conical shape and the indented surface^15,17^. The total force applied by each conical element then becomes:

9 $${F}_{0}=\frac{2{E}_{t}^{\ast }{\delta }^{2}}{\pi \,\tan \,\theta },$$

and the mean contact pressure for each element is defined as:

10 $$\bar{P}=\frac{1}{2}{E}_{t}^{\ast }\,\tan \,\theta .$$

### Measurement of arterial drug transfer

Stored arterial vessel samples (obtained after mechanical testing) were prepared for high-performance liquid chromatography (HPLC) to measure the amount of transferred drug. Each sample was first submerged in methanol and vortexed briefly. The vessel was then homogenized in methanol for 3 minutes using a probe sonicator and vortexed again for 10 minutes. The sample was then centrifuged at 1000 rpm for 10 minutes. The supernatant holding the extracted drug was transferred to a fresh experiment tube. This sample was diluted in methanol and readied for HPLC testing. The extraction method above was validated by two control experiments; dissolving known amounts of drug in methanol and loading drug (paclitaxel) onto vessel samples followed by rapid freezing, extraction using methanol and dilution prior to HPLC. Then, standard solutions and sample solutions were analyzed through HPLC.

### Cell culture

We performed cell culture experiments to determine if the presence of different balloon coating materials would affect cell viability. Human umbilical endothelial cells were cultured as described previously^18^. Cells were maintained at 37 °C and 5% CO_2_. Early passage cells (less than eight passages) were used for the study.

### Flow cytometry

To examine the cytotoxicity of the excipients on live cells, LIVE/DEAD staining followed by analysis was conducted. Endothelial cells (2 × 10^5^) were plated in 12 well plates and were treated for 24 hours with a titrated concentration of the shellac and urea. Molecular grade ethanol (Pharmco) was used to dissolve shellac (Sigma Aldrich) (10 µM–100 mM) and double distilled autoclaved water was used to dissolve urea (Sigma Aldrich) (10 µM–100 mM). Single cell suspensions were analyzed using FACS LSR II (BD Biosciences). Cells were harvested and viable versus non-viable cells determined by staining with Zombie UV™ Fixable Viability Kit (Biolegend), as described previously^19,20^. Cells were stained in dark conditions for 30 minutes at room temperature in protein free phosphate buffered saline. Gating was done on live cells whose intact membranes prevent dye infiltration while dye positive dead cells were excluded. The positive control consisted of dead cells, which were induced using Staurosporine^21^. Data were analyzed with Flowjo software (Tree Star).

### Cell viability assay

To examine the cytotoxicity of the excipients, viability of the cells was determined by measuring released ATP using the Cell Titer–Glo Luminescent assay (Promega) performed using manufacturer’s instructions. Endothelial cells (5 × 10^4^/100 µL) were plated on a 96 well plate and treated with various concentrations of 200 proof ethanol (Pharmco) dissolved shellac (10 µM–100 mM) and double distilled autoclaved water dissolved urea (10 µM–100 mM) for 24 hours. Cell Titer Glo buffer and substrate were equilibrated to room temperature. The buffer was added to the lyophilized substrate and mixed gently using vortexing. Equal volume (100 µL) of the homogenous solution was added to the plated cells. Luminescence was measured with a plate reader. Luminescent signal is proportional to the amount of ATP. Presence of ATP is directly proportional to the number of viable cells in culture. Data were analyzed with GraphPad Prism.

### Statistical analysis

Descriptive statistics are presented as the mean and standard deviation. Unpaired Student’s t-test was used to compare experimental groups as appropriate. For some cases, significance of the Pearson correlation coefficient was computed using two-tailed probability, given the correlation value (R^2^) and the sample size. A p-value less than 0.05 was considered statistically significant.

## Results

### Surface morphology of the coatings

SEM imaging allowed us to characterize the distinct morphologic structures of PTX-urea and PTX-shellac, respectively (Fig. 3A,B). We performed image processing on the SEM images to measure the intrinsic shape and the number of microstructures per unit area of the coating microstructures. The shellac coating consisted of spherical microstructures with a mean radius of 0.84 µm and a standard deviation of 0.30 µm (Fig. 3A), and the microstructures distributed densely with 0.18 microstructures per square micrometer. The urea coating consisted of needle-like cyrstalline mircostructures with a mean length of 21.6 µm and a standard deviation of 8.5 µm (Fig. 3B), and the microstructures distributed more sparsely with 0.003 microstructures per square micrometer. As these surface morphologic signatures can lead to dissimilar modes of interaction with the arterial vessel, we hypothesized that these modes can also lead to differential drug transfer patterns. Additionally, the Hertz theory allowed us to relate SEM-observed intrinsic shapes of the coatings to define the contact regions between the balloon coating and the vessel wall.

### Mechanical properties of the test elements

We leveraged uniaxial compression testing to simulate the coating-artery interaction during angioplasty. The compression phase of the experiment simulates balloon inflation, which was reflected as a gradual increase in tissue displacement with increasing applied compressive force. The compressive moduli of the test samples were calculated from the force-displacement (FD) curves during the compression phase. During the 60 s displacement dwell period, the test constructs underwent a relaxation phase, as indicated by the gradual reduction in compressive force. During the decompression phase, the coating is separated from the contact region and the applied force gradually returns to zero, indicating complete interfacial failure, which could be attributed to adhesive failure of formed interface and/or fracture of the balloon coating (Fig. 1B).

**Figure 4:**
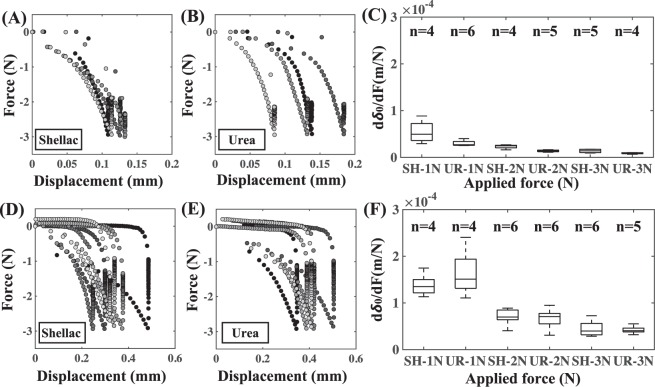
Bulk interfacial mechanics are independent of excipient type. Force-displacement (FD) curves from the mechanical test when shellac (**A**) and urea (**B**) were used as the balloon coating materials, respectively. For (**A**,**B**), the test element comprised of the balloon coating compressed on a flat, rigid surface, which allowed us to estimate the balloon compliance (**C**) of shellac (indicated by the abbreviation SH) and urea (indicated by the abbreviation UR), respectively. FD curves from the mechanical test when shellac (**D**) and urea (**E**) were used as the balloon coating materials, respectively, where the test element comprised of the balloon coating compressed on an excised porcine arterial vessel. This experiment allowed us to estimate the overall compliance values of the 2-element system (**F**) with shellac and urea, respectively. The dwell time was maintained at 60 s with a maximum application force of 3N.

The first set of mechanical testing experiments generated FD curves during the compression phase when shellac- and urea-coated balloons were compressed on flat, rigid surfaces, respectively (Fig. 4A,B). These experiments allowed us to estimate the mean compliance values of both the urea- and shellac-coated balloons at the maximum compressive force of 1, 2 and 3N, respectively (Fig. 4C). Interestingly, there were no statistically significant differences between the mean compliance values of shellac- and urea-coated balloons (p = 0.36 for 1N, p = 0.41 for 2N and p = 0.62 for 3N). The second set of mechanical testing generated FD curves during the compression phase when shellac- and urea-coated balloons were compressed on excised femoral arteries opened *en face* so that the intraluminal side of the vessel was in contact with the balloon coating (Fig. 4D,E). Mean overall compliance values of the 2-element test constructs were then computed (Fig. 4F). Even for this set of experiments, we found no statistically significant differences between the mean overall compliance values of shellac and urea (p = 0.27 for 1N, p = 0.87 for 2N and p = 0.84 for 3N). Later, for each experiment that generated a unique FD curve during the compression phase, the compliance of the arterial vessel used for that experiment was then computed as the net difference between the overall compliance of the 2-element system and the estimated average compliance of the coated balloon. In this fashion, we were able to compute arterial sample-specific estimates of compliance, and these values were used further to quantify the coating-artery interactions. Taken together, these results imply that within our experimental design space and under the examined mode of coating-tissue interaction, bulk interfacial mechanics are insensitive to excipient type.

### Relating coating-specific interactions with arterial drug transfer

**Figure 5:**
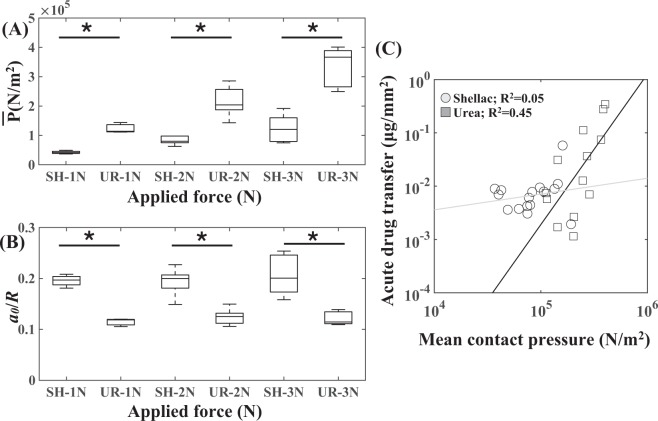
Excipient microstructure modulates acute transfer of balloon coating. Mean contact pressure was computed by normalizing the application force with the intrinsic shape of shellac (**A**) and urea (**B**). Normalized contact radius was different between shellac (**C**) and urea (**D**) as well. Asterisks indicate statistical significance (p < 0.05). (**E**) Net coating transfer as a function of mean contact pressure for urea and shellac. Data on both axes is plotted in log-10 scale.

We hypothesized that coating surface microstructure impacts the interactions between the coating and the arterial wall. We computed the mean contact pressure for each coating, which is a function of the applied force and the contacting surface area of the coating (Fig. 5A). Interestingly, we found statistically significant differences in the mean contact pressure values between shellac and urea as a function of application force (p = 1.2e-2 for 1N, p = 3.3e-3 for 2N and p = 7.6e-4 for 3N). We also found that the size of the contact region was different between urea and shellac, respectively (Fig. 5B), regardless of the amount of application force (p = 1.0e-4 for 1N, p = 6.0e-4 for 2N and p = 2.2e-3 for 3N). Importantly, acute drug transfer following mechanical testing correlated with mean contact pressure in a coating-specific fashion (Fig. 5C; R^2^ = 0.45, p = 1.3e-2 for urea and R^2^ = 0.05, p = 0.465 for shellac). These results indicate a stronger correlation of drug uptake with mean contact pressure in the case of urea than for shellac. Systematic analysis in this fashion can allow us to quantify these relationships to a broad range of candidate excipients.

### Cytotoxicity of the excipient

**Figure 6:**
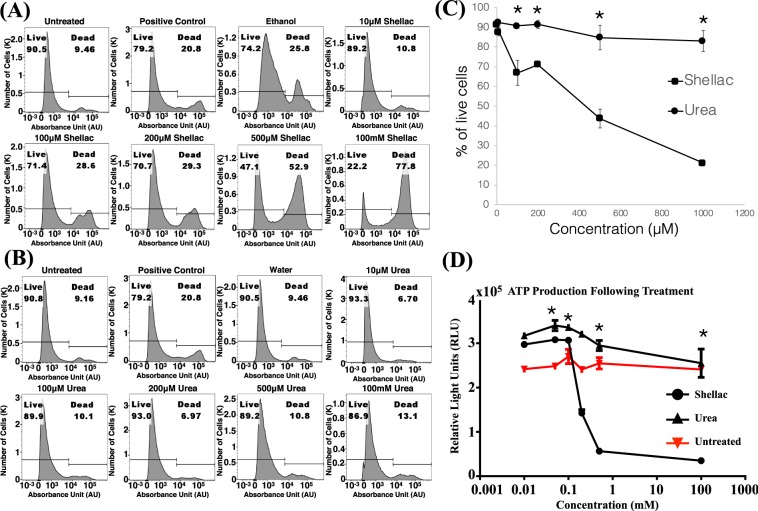
Cytotoxicity of the excipients. (**A,B**) Cell death with increasing concentrations of shellac and urea. Endothelial cells (1 × 10^6^) were treated for 24 hours with increasing concentrations of shellac and urea. Cells were harvested and stained with Zombie UV™ viability fluorescent dye and analyzed by flow cytometry. Heat-shocked cells were used as positive control. Ethanol was used as a vehicle control for shellac and water for urea. Representative FACS images from two independent experiments is shown. Increase in concentration of urea did not result in decreased cell viability. (**C**) Average percentage of live cells in response to the treatment of shellac and urea done in two independent experiments is shown. The symbol * indicates p-value that compares live cells between shellac and urea-treated samples. Also, p = 0.03 for 100 µM, p = 0.005 for 200 µM, p = 0.017 for 500 µM, p = 0.004 for 1000 µM. Standard errors are shown on error bars. (**D**) Average of ATP production from two independent experiments is shown. ATP production indicates the viability of endothelial cells as a function of increasing concentrations of urea and shellac. The symbol * indicates p-value that compares ATP between shellac and urea-treated cells. Also, p = 0.02 for 100 µM, p = 0.001 for 200 µM, p = 0.001 for 500 µM, p = 0.01 for 1000 µM. Increase in concentration of urea did not compromise ATP production in endothelial cells. Standard errors are shown on error bars.

While the coating shape-dependent aspects associated with corresponding transfer of the balloon coating, the task was to determine how these coatings behaved within the cellular environment. As such, DCBs are placed within the vessel wall, which exposes the coating directly to the vascular endothelium. To this end, human umbilical endothelial cells were exposed to titrated concentrations of shellac and urea for 24 hours. The cells were subjected to LIVE/DEAD and ATP release assays, respectively. Both these assays showed that low concentrations (10 µM/mL) of shellac and urea did not affect cell viability compared to vehicle treated cells (Fig. 6A,B). However, shellac from 10 µM to 100 µM resulted in an increase from 8.5% cell death to 30.5% cell death. This change was not found in similar concentrations of urea. Increasing the concentration of urea from 10 µM to 100 µM, did not affect cell viability and this trend continued up to a 10,000-fold increase of urea to 1 mM. These data corroborated with the luminescent cell viability assay (Fig. 6C). Shellac demonstrated a dose-dependent decrease in cell viability. Cells treated with higher dosages of shellac resulted in decreased ATP levels when compared to cells treated with the same concentration of urea (Fig. 6D). Since the presence of ATP is an indicator of metabolically active cells, this assay strongly suggests that shellac potentially decreases cell viability at high concentrations. Collectively, both these assays consistently showed lower viability at an eqimolar concentration of shellac compared to urea.

## Discussion

Catheter-based endovascular therapy involving balloon angioplasty in concert with local drug delivery is highly appealing as it circumvents the use of permanent indwelling implants such as stents^9,22–27^. Balloon angioplasty alone or angioplasty followed by stenting as such have stood the test of time^28^, and their strengths as well as limitations are appreciated by the clinical community. On the other hand, clinical studies focused on intraluminal delivery of therapeutic compounds from these balloon catheters have demonstrated great promise but have not yet fully proven to result in a sustained, long-term benefit^6,29,30^. Balloon-vessel contact times are short due to the nature of the procedure (2–3 min), and moreover, the process of coating transfer from the balloon to the vessel wall and drug delivery efficiency within this period are not fully understood. Continuation of this trend can lead to a problem - DCB therapy could remain under-appreciated and interventional strategies for PAD using currently approved DCBs can lead to sub-optimal outcomes. It is therefore important to identify factors that promote efficient delivery and track consequent arterial pharmacokinetics.

Using a series of bench-top experiments and modeling the interaction between the balloon coating and the arterial wall (Fig. 1), we computed compliance values of the arterial wall and mean compliance values of the balloon coating. Using the classic Hertz theory, we defined shape-specific contact mechanics and the contact force applied on the arterial wall. Mean contact pressure, which is a function of the contact force and the intrinsic shape of the coating, was then computed for each of these cases and associated with corresponding acute transfer of the balloon coating (Fig. 5E). Also, endothelial cells exposure to either shellac or urea, induced differential toxic effects that were dose-dependent (Fig. 6). These results underscore the importance of fully characterizing the nature of the excipients so as to optimize DCB therapy.

The DCB excipient has an important role to play before, during and after balloon angioplasty. During the pre-procedural or the design phase, a coating technique is used to create a thin layer on the surface of the balloon, where the excipient directly plays a role to support adhesion of the coating to the balloon. During the procedure, as the balloon is expanded to reopen an occluded arterial vessel, a portion of the balloon-adhered drug coating detaches from the balloon and gets transferred to the arterial wall. Here, the excipient acts as the carrier to facilitate rapid drug transfer to the mural surface. The procedural time is short (~2–3 minutes) but can be logically considered as 2 sub-phases defined as the compression and decompression of the tissue-balloon interface (Fig. 1B). During the compression sub-phase, the coating is adhered to the balloon and concomitantly initiates contact with the mural surface. This mechanical contact initiated due to the radially outward force generated by balloon expansion leads to adhesion of the coating to the mural surface. Several properties of the excipient play a role in terms of dictating the extent of adhesion. During the decompression sub-phase, the radially inward force generated by balloon deflation generates interfacial failure between the mural surface and the drug coating and potentially between the balloon and the drug coating. Cohesive bond failure within the coating itself is also a possibility during the decompression sub-phase. Once the procedure is completed, the expectation is that the transferred drug coating continues to adhere to the mural surface, which can lead to a sustained therapeutic response. In this phase, the excipient acts as an agent to support adhesion and creates a source for local delivery of drug to the mural surface. In sum, the pre-procedural design considerations, the procedural aspects involving different modes of bonding failure and post-procedural phenomenon related to mural adhesion of drug coating can together determine the success of DCB delivery. As a first step towards fully characterizing the role of excipients, we focused on quantifying the impact of coating surface morphology and its interaction with the mural surface during the procedure on acute transfer of the balloon coating.

Our study’s main finding is that the unique microstructure of the balloon coating can directly impact acute transfer of the balloon coating. This is due to the fact that at this scale, the interaction between a spherical-shaped element and the arterial wall is fundamentally different from that of a conical-shaped element, even when the same external force is applied by the balloon on the arterial wall. The classic Hertz theory allowed us to mathematically decompose this deterministic aspect of contact mechanics (Fig. 3C,D) in terms of the mean contact pressure associated with a specific excipient and application force (Fig. 5A,B). We found distinct differences in balloon transfer of paclitaxel as a function of mean contact pressure for urea and shellac (Fig. 5E).

Our study has some limitations. We used explanted porcine femoral arteries for mechanical testing and for subsequent measurements of drug transfer. These vessels may lose some structural integrity immediately after sacrifice, even though we took utmost care to preserve tissue viability. Our uniaxial mechanical testing protocol is designed to isolate contact phenomena that likely underlie DCB performance, but does not mimic exact clinical deployment of these devices. We assumed linear elastic behavior for all components in our contact mechanical model, although nonlinear effects may become important at larger strains/strain rates experienced in device deployment. During mechanical testing, both cohesive and adhesive bond forces play a role during compression and decompression phases, respectively, and this dynamic may ultimately determine the amount of drug coating adhered to the vessel wall. We did not attempt to isolate the individual roles of cohesion and adhesion in the context of our study, as our goal was to estimate metrics relevant to balloon-tissue contact and acute drug transfer. This type of bonding failure (adhesive or cohesive) will dictate the amount of drug coating that is retained on the mural surface under physiologic conditions (i.e. blood flow), and is thus significant to drug retention at the application site.

In conclusion, our study connects the application force, which is a procedural factor that exemplifies balloon angioplasty with the intrinsic shape of the balloon coating microstructure, which is a design parameter of the device itself. By relating the coating-specific morphology and its subsequent interactions with the arterial vessel, we have demonstrated a mechanistic basis by which to evaluate different balloon coatings. While examination of bulk interfacial mechanics found no coating-specific differences, mean contact pressure computed by normalizing the application force with the intrinsic shape of the coating was significantly different between urea and shellac. This means that no two excipients can be alike in terms of their microscopic mechanical interactions with the arterial vessel, and when coated on the balloons along with the same drug can still result in differential arterial pharmacokinetics. Our paradigm can be further used to design coating formulations that can have maximal balloon transfer of drug, higher arterial uptake and minimal loss within the systemic circulation, thereby paving way towards efficient therapeutic approaches using balloon catheters.
